# Report of the Special Committee of the American Medical Association, upon Medical Education

**Published:** 1858-07

**Authors:** 


### EDITORIAL AND MISCELLANEOUS.

### Report of the Special Committee of the American Medical Association, upon Medical Education.

At the meeting of the American Medical Association, held in Nashville, in May, 1857, *two* most unprecedented acts of discourtesy were committed by the *controlling* influence in that body: 1st. The appointment of a *Special Committee* upon the *subject* of Medical Education, thus susperceding the *standing committee*, upon the same subject, and thus expressing in the most unequivocal manner, a want of confidence in the latter. And, 2dly, in not only failing to appoint the mover of the resolutions, (Dr. Currey,) for the consideration of which the Special Committee was constituted, as the Chairman, but in addition, actually excluding him entirely from it.

As the result of this course of proceeding, we have the Report made at the late meeting of the Association, by Dr. Jas. R. Wood, of New York, certain points of which, we unhesitatingly declare our belief, to have originated with the University of Nashville, with the view of breaking down a troublesome competitor—the Atlanta Medical College.

Without going, at present, into a *detail* of the reasons for this conclusion, we would state, that this Institution had good reason previous to the meeting of 1857, (already referred to,) to *know* that it was the intention of the Nashville School, to make an effort to exclude her from the Association, having already attempted to discredit her with the profession.

This effort *was made* by (as we believe) *procuring* the introduction of a set of resolutions, the object of which was to exclude any Medical Institution from representation in the Association, where a student was graduated without an interval of six months between the courses of lectures which he had attended, though he might have been engaged in the study of

Medicine, not only three years, but an unlimited period, when at the same time, they were notoriously disregarding not only *time* but *qualification*, in their requisitions for graduation.

It is therefore, *not true*, as stated in the report of the Special Committee, that "at the late meeting of the Association, at Nashville, the subject of Medical Education was again *introduced** in a series of resolutions, denying that this Association has the power to control the subject of Medical Education, and deprecating any further agitation of it in this body." Such *resolutions were introduced*, but not until the subject of Medical Education had been "thrown (as a distinguished member of the Association remarked, who fully understood, and who had the best opportunity of knowing, *the object*,) as a firebrand into the Association."

The resolutions denying the power to the Association to *control* the subject of Medical Education contained nothing but the *truth*, and scarcely more than is admitted in this same *Special Report*, that "thus far, little has been accomplished but discussion and agitation. The Schools have been indisposed to adopt the proposed changes in their curriculum of studies, and *this Association has lacked the power to render obligatory its proposed reform.*"

But notwithstanding, this distinct and positive admission of the inefficiency of the Association in regulating this matter, and that its discussion had been mainly a source of discord, we have it declared in the same breath, that "it exercises by its moral force, a compulsory power."

We admit that the American Medical Association may regulate its own members, according to certain conditions and stipulations upon which they entered the body, and in addition, may and does exert a *moral influence* upon the Profession at large, but we cannot conceive any greater absurdity, than the idea of a "compulsory power," above and beyond rights and powers which have been *expressly granted*, and from which *alone* any controlling influence is derived. .The Association has accomplished much for the Profession, and by confiuing

* Italics are ours.

itself within its legitimate sphere, may accomplish much more. It *has* a "moral power" which should be exercised with great care on account of its very influence—to be a true "moral power," however, it must have *justice* as one of its most important elements.

What does this report, which contends so strongly for this "moral power" of the Association, propose to do? We say unhesitatingly that in a prominent feature, it is but the embodiment of the plans and purposes of one portion of the Association to destroy another. Originating in a meeting of the Association *known* to have been controlled by the Nashville Medical School, and bearing unmistakeably the *impress* of its designs, it *proposes* to become the instrument of the grossest *injustice.*

We do not care to say that this feature was *dictated* directly by those with whom it *originated*, but we do say that it is only the execution of a purpose formed before, and developed at, the meeting of the Association in Nashville, to use the power of this body, to sap the foundations of a troublesome competitor, whose success was supposed to be dependent upon the *season of the year*, at which its sessions were held.

Again, what does this report, which echo's so strongly the views of the late retiring President, in reference to the "moral power" of the Association—himself, a member of the faculty of the Institution, to which we have alluded—propose to do? It proposes to adopt a *system* of Medical Education, which would throw discredit upon Institutions equally respectable where they are located, with those of any other section, the objectionable feature of the "system," at the same time, being based upon a *simple absurdity*, "that medicine can not be taught in the *summer*," and thus proposes to become the instrument of injustice and oppression, and the violator of sacred rights and privileges.

Again, we take issue with this report in its statement, as to the American Medical Association constituting a "National Congress," which of right "exercises an influence, and wields a power that has all the force and energy of a government,"

this claim being based, according to the author of this paper, "upon the consent of the governed, and on the equitable principle, that, in representative bodies, the majority must rule."

Take for instance the last meeting of the Association, at Washington City, the most National meeting ever held, and what do we find—New York, Pennsylvania and Massachusetts, constituting at least one-half of the whole body, and possessing unquestionably the power to use the Association at pleasure. Has the profession of all the other States *consented to be governed* by the profession of these States, or of any others who *happened* to be in the ascendancy, in the Association. The body never can be a full and fair representation of the medical men of the United States, as in all its meetings, it will ever be made up to a large extent, of those who are most convenient, or who have the best facilities for reaching the place where it is held.

The South and West have taken comparatively little interest in the annual convocations of this body, the former, more particularly, may be said never to be *represented*, a number of the Southern States having no delegates in the last meeting. There is, therefore, no consent of the medical profession of the UnitedStates to be *governed* by the American Medical Association, and nothing upon which to found "the equitable principle belonging to representative bodies, that the majority must rule," and consequently no justice or propriety in the assumption that a majority in this body have at any time a right to *rule* in matters affecting the rights and interests of the mass of the profession, throughout this vast country.

Surely, New York and Pennsylvania, constituting nearly one-half of the late Association, will not say to us in Georgia, or Vermont, you shall not establish an Institution for the instruction of your own people in Medicine, except upon conditions prescribed by us; we claim the privilege in Georgia, at least, of organizing our own Institutions, without reference (as a matter of obligation) to those of other States. We get no patronage from the North—their Institutions have been largely built up by Southern contribution, which will doubtless to a considerable extent, in the folly of our own people, continue to be poured into their coffers, and we therefore claim the poor privilege of being allowed to educate *a portion* of the people, born upon our own soil, upon a plan that suits ourselves. Let each State regulate its own internal affairs, without interference from without, and if it is really the idea to have a National Association, ever respect the *rights of the States, in giving to the Profession of each individual State, the right and power of saying, who shall be recognized as medical men.* The American Medical Association will be far below what should be its high aims, when allowing itself to be used as an instrument of cliques or colleges to bring discredit upon any portion of the profession who are holding the same great truths, and have the same high objects in view, and in such a vocation, will assuredly find a speedy termination to its career.

We are honestly desirous to elevate the standard of medical education, and will ever be found uniting in any legitimate and practicable plan for this object. We are especially willing at all times to submit to the will of the masses of the profession, (as expressed through our local and State Societies) who can alone be competent judges of a system of education which is adapted to their own sections; but we have never yet brought ourselves to the conclusion, to submit to the *intrigues* of selfish competitors.

But it may be said that this report *allows* the "Medical Colleges an opportunity to consider the recommendations here advanced before any definite action is taken upon them;" to which we would reply, that this is poor consolation to those, upon whom an attempt has been made to cast an odium, and who would therefore go into a Convention under the most disadvantageous circumstances. If a Convention of the Colleges was the remedy for the difficulty, and if "their wisdom and mature views," were so important to a correct understanding and a proper arrangement of this subject, and if that harmony and concert action—from which alone any good could arise—was desired, why, we ask, make a suggestion striking at the very foundation of a portion of the schools, without giving any thing which could bear the name of a reason, for such recommendation? Because, we reply, it was a foregone conclusion, so far as this particular point of the report was concerned, and as such, entitled to no respect.

But to leave this feature of the report which deserves and receives our most unmitigated contempt, we would turn to another which meets our most unqualified approbation, and which *alone*, would have made a much more creditable report, than that which has been so elaborately prepared.

It is proposed to remove the diploma-granting power from the schools, and place it in the hands of an examining Board, the members of which shall have no College appointments. In this direction, as we have intimated upon a former occasion, lies the true remedy for the present admitted defects in our plan of Medical Education.

But while we admit that an improvement may be made in medical education, and that unqualified men enter the profession, we do not believe in the truth of the *stereotyped cry* of the inferiority of the Medical Profession of the United States—the forthcoming volume of the transactions of the American Medical Association, is of itself sufficient to brand it as slander. It has been too long the habit to decry our profession in this country, and to make invidious comparisons between the American and the European physicians, greatly to the discredit of the former—of this we are heartily tired, and claim for the regular medical men of our country, a full equality with those to be found any where, in practical knowledge and skill in the treatment of diseases, with which they have to contend. In conclusion of this desultory review of the report of the Special Committee upon medical education, before the late meeting of the American Medical Association, we would remark, that we have not intended, nor do we feel any special disrespect for its author, though believing, as already distinctly stated, that he has been made the tool (perhaps unwittingly) of designing men, who are looking more to the advancement of selfish purposes than to that of medical education.

If we believed that the present movement upon the subject of Medical Education, had arisen from a sincere desire to elevate the profession, we should unquestionably take a different view of the whole matter, and might be disposed to submit to many things which we should now scorn to consider, believing this as we do, only another mode of accomplishing a selfish and long cherished end, after every other has failed. We wish it, however, distinctly understood, that we speak for ourselves alone—what course the Institution with which we are connected may choose to pursue, we do not pretend to indicate—it may be represented in the Convention, and may find some acceptable system of medical education presented, but will unquestionably never submit to the *dictation* of those, whose *interest* it is, to change her policy.

There are other suggestions and recommendations in this report, which we regard as illiberal, visionary, and impracticable, to which, however, we do not propose giving any attention at present, farther than to remark, that it is perfectly manifest, that a strong effort is about to be made to sap the foundations of all medical schools, save those which are found in the larger cities, in the contest arising out of which, we shall confidently appeal to the masses of the profession, to stand up against the *dictation* and grasping selfishness of a *few* would-be "regulators" of medical affairs in the United States, who affect to have a supreme contempt for all medical education not obtained in certain Schools or *Hospitals* with which they happen to be connected.

